# Tracheal remodelling in response to hypoxia

**DOI:** 10.1016/j.jinsphys.2009.05.008

**Published:** 2010-05

**Authors:** Lazaro Centanin, Thomas A. Gorr, Pablo Wappner

**Affiliations:** a Institute of Zoology, Im Neuenheimer Feld University of Heidelberg, 69120 Heidelberg, Germany; b Institute of Veterinary Physiology, Vetsuisse Faculty and Zurich Center for Integrative Human Physiology (ZIHP), University of Zurich, Wintherthurerstrasse 260, CH-8057 Zurich, Switzerland; c Instituto Leloir and FBMC, FCEyN-Universidad de Buenos Aires, CONICET, Patricias Argentinas 435, Buenos Aires 1405, Argentina

**Keywords:** Tracheae, Plasticity, Hypoxia, HIF, Cell autonomy

## Abstract

The insect tracheal system is a continuous tubular network that ramifies into progressively thinner branches to provide air directly to every organ and tissue throughout the body. During embryogenesis the basic architecture of the tracheal system develops in a stereotypical and genetically controlled manner. Later, in larval stages, the tracheal system becomes plastic, and adapts to particular oxygen needs of the different tissues of the body. Oxygen sensing is mediated by specific prolyl-4-hydroxylases that regulate protein stability of the alpha subunit of oxygen-responsive transcription factors from the HIF family. Tracheal cells are exquisitely sensitive to oxygen levels, modulating the expression of hypoxia-inducible proteins that mediate sprouting of tracheal branches in direction to oxygen-deprived tissues.

## 1 Introduction

Life maintenance demands an enormous amount of energy, and among the stable chemical elements existing on earth, oxygen reduction provides the largest possible release of free energy. Animals have developed during evolution relatively complex systems to capture and deliver this precious molecule: Lungs and blood vessels in mammals, and the tracheal system in insects constitute two examples of these successful adaptations. On top of transporting oxygen from the atmosphere to the cells, in order to cope with environmental variations, delivery systems have to adapt to oxygen availability. We will review here how the tracheal system of *Drosophila melanogaster* responds to hypoxia, focusing on the ability of the tracheae, and particularly, of a specific cell type called tracheal terminal cell (TTC), to respond autonomously to variations of environmental oxygen tension. TTCs can sense hypoxia and respond by producing extra-ramifications, and this behavior shares many features of that of the “tip cell” of growing blood vessels during mammalian angiogenesis.

## 2 The intrinsic plastic—nature of oxygen delivery systems

Metabolic rates increase with animal size. Since size is gained more by volume (∝ to *r*^3^; *r* is radius) than surface (∝ to *r*^2^), the surface/volume ratio declines with larger body size and, thus, bigger animals demand more efficient oxygen supply systems (White et al., 2003). This simple rule seems to apply throughout evolution. In fact, the geologic event known as ‘The Big Oxidation’, during which a major rise in oxygen levels occurred at the early Cambrian Period, has been the driving force for the appearance of animals with more complex body plans (Valentine, 1994). An atmosphere with high oxygen levels fostered the evolution of efficient oxygen delivery systems. It also triggered the emergence of mechanisms that allowed utilization of energy derived from complete oxygen reduction, while parallel antioxidant reactions were set in place to avert the accumulation of damaging reactive oxygen species (ROS) stemming from incomplete reduction of O_2_. These evolutionary acquisitions towards a safe and efficient metabolization of oxygen contributed to a large extent to the “Cambrian Explosion”, an inflexion point of our geological history in which morphological body plans reached unparalleled rates of diversification (Valentine, 1994). In addition to promoting morphological diversification, oxygen delivery organs have been essential for animals to attain bigger sizes during evolution. This is clearly illustrated by the fact that severe or prolonged impairment of the system responsible for oxygen supply leads to animal developmental arrest and death (Carmeliet et al., 1996; Carmeliet and Collen, 2000). In line with this, growth of solid tumors is tightly related to oxygen supply through angiogenesis: The growing mass of cells in a tumor generates local hypoxia, which, in turn, leads to recruitment of new blood vessels. Newly formed blood vessels re-supply blood born nutrients and oxygen to support further growth of the tumor (Liao et al., 2007). Without angiogenesis, the growth of many tumors will not exceed the size limit imposed by oxygen diffusion from existing capillaries. A great deal of effort is being currently invested to intend interfering with tumor-driven angiogenesis.

In insects, the tracheal system – a network of air-filled tubes that reaches virtually every cell in the organism – mediates delivery of oxygen gas to organs and tissues throughout the body (see below). Given that insect development typically involves major changes of body general architecture and size, the tracheal system faces a major challenge, as it has to be plastic enough to adapt to such changes. Insect life cycle typically comprises an embryonic development in the egg, followed by larval stages in which the insect feeds very actively, and dramatic body growth occurs. For example, in *Drosophila melanogaster* the larva increases its volume more than 200 times in 3 days; in *Manduca sexta* and *Bombix mori* body weight increase is even larger (1st Instar larvae are around 1–2 mm long, and 5th Instar larvae can reach up to 6 or 10 cm in length respectively). Thus, larvae need a protein-rich diet together with an efficient oxygen delivery system. In addition, the respiratory organ needs to rapidly adapt and respond to increasing oxygen demands, derived from the dramatic rate of growth of the larva. Whereas in hemimetabolous insects the adult stage is attained after several larval molts without substantial alteration of the general body plan, in holometabolous insects, the overall body plan is greatly modified during the pupal stage that follows larval development and precedes the adult stage. Therefore, in holometabolous insects, such as the fruit fly *Drosophila* *melanogaster*, the tracheal system faces the extra-challenge of undergoing a profound remodeling to cope with the change in body plan. Here in particular, the plasticity of the tracheal system is critically important to sense changing levels in oxygen partial pressure (*p*O_2_) in the tissues, and respond by adjusting its extent of growth and ramification to regulate oxygen supply. In the next sections we will briefly describe some features of the development of the *Drosophila* tracheal system, and focus on its ability to undergo morphological adaptations in response to *p*O_2_.

## 3 Development of the tracheal system

Unlike vertebrates, which have developed two different but still coupled systems for oxygen intake and distribution – i.e., lungs or gills for oxygen intake, and a circulatory system for oxygen delivery to tissues and organs – insects rely solely on the tracheal system, an interconnected air-filled network of tubes that transports oxygen in gas phase directly to tissues and cells throughout the organism (Ghabrial et al., 2003).

Our understanding of the genetic control of insect tracheal development is mainly based on the work carried out in *Drosophila* *melanogaster.* The development of *Drosophila* tracheal system begins around 4 h after oocyte fertilization, to form an interconnected network of tubes during embryogenesis. This network becomes functional at the beginning of the first larval instar and ramifies dramatically during the 2nd and 3rd larval stages to accompany the increase in body size of the larva. Finally, it is rebuilt at the pupal stage to acquire the ultimate pattern of the adult (Ghabrial et al., 2003; Weaver and Krasnow, 2008).

The tracheal system arises at mid-embryogenesis as 10 independent ectodermal placodes of around 80 cells each at either lateral side of the embryo (Samakovlis et al., 1996a). These flat placodes can be initially identified by the expression of gene *trachealess*, which encodes a basic Helix–Loop–Helix-Period Arnt Single-minded (bHLH-PAS) transcription factor that controls transcription of downstream genes that mediate tracheal development (Isaac and Andrew, 1996; Wilk et al., 1996). Each placode then invaginates to form a sac, and immediately afterwards, tracheal cells migrate in highly stereotyped directions to give rise to six tracheal branch primordial (Vincent et al., 1997; Wappner et al., 1997). After tracheal branches have extended, most of them meet and fuse to branches arising from the contralateral hemisegment, or from the adjacent segments, to generate an interconnected and continuous network of epithelial-like tubes (Samakovlis et al., 1996b). The Fibroblast Growth Factor Receptor (FGFR) pathway plays a cardinal role in the process of guided cell migration that accounts for the initial branch formation and extension (Reichman-Fried et al., 1994; Sutherland et al., 1996). All tracheal cells express the FGFR homologue Breathless (Btl), via the transcriptional control by Trachealess (Glazer and Shilo, 1991; Klämbt et al., 1992; Reichman-Fried and Shilo, 1995). Outside the tracheae, in the target tissues, the FGF homologue Branchless (Bnl) acts as a chemo-attractant for migrating tracheal cells (Sutherland et al., 1996). Budding tracheal branches that express Btl migrate towards clusters of cells expressing the ligand Bnl. Once tracheal cells have reached the Bnl-positive cluster, *bnl* expression shuts-off in that cluster, and is immediately turned-on again a few cell diameters away on the track of the forming branch. This process repeats again and again throughout branch extension, promoting the continuous elongation of the tracheal branches (Sutherland et al., 1996).

On top of accounting for tracheal cell migration, the FGFR pathway plays two additional roles during tracheal development.

### 3.1 FGFR pathway activation leads to the differentiation of one of the cells of the forming branch into a “terminal cell” fate

The tracheal terminal cell (TTC), also known as tracheolar cell (Chapman, 1998), is the only tracheal cell that has the capacity to produce cytoplasmic extensions (i.e. “terminal branches” or “tracheolar branches”) to deliver oxygen to target tissues (Harrison, 2003). Cells at the tip of each branch acquire a terminal cell fate during tracheogenesis (Affolter et al., 1994; Guillemin et al., 1996). Remarkably, experiments carried out in the dorsal branch (which possesses just one terminal cell) revealed that the cell with the highest level of FGFR pathway activation will outcompete the other cells of the branch with lower levels of FGFR activation, to become “the” terminal cell (Ghabrial and Krasnow, 2006). When FGF signaling is artificially increased in the entire tracheal tree, either by over-expression of Bnl or by expression of a constitutively active form of Btl, multiple ectopic terminal cells arise in each branch (Sutherland et al., 1996). TTC fate is usually assayed either by morphology or by expression of the Serum Response Factor (dSRF), a terminal cell marker and key molecule regulating the cytoplasmic changes during TTC branching (Reichman-Fried and Shilo, 1995; Guillemin et al., 1996; Lee et al., 1996).

### 3.2 The FGFR pathway modulates the extent of ramification of tracheal terminal cells

Augmented FGFR activation increases the ramification of terminal tracheal cells, whereas reduction of FGFR activity, once the tracheal branches have reached their final destination, leads to reduction of terminal cell sprouting (Hacohen et al., 1998).

## 4 Oxygen availability modulates the branching of tracheal terminal cells

**Fig. 1:**
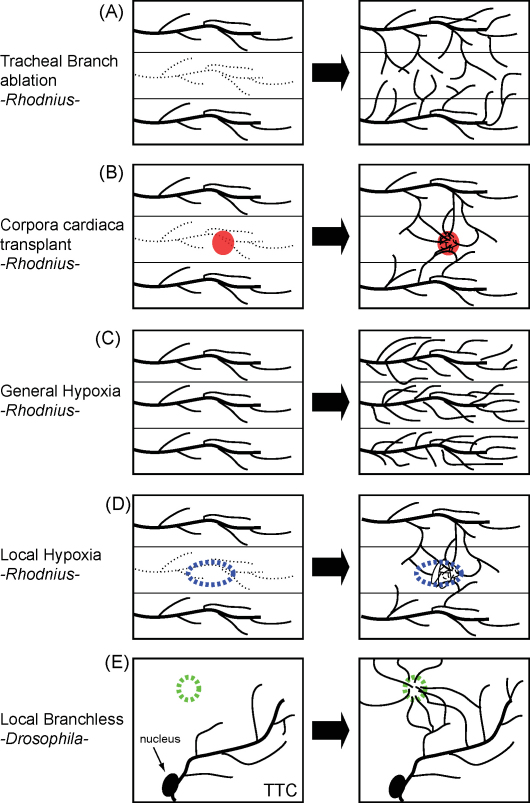
Insect terminal tracheal cells ramify in response to hypoxia. (A) A tracheal branch corresponding to one of the *Rhodnius* abdominal segments was surgically severed (dotted line in the left panel). A few days later, tracheal branches arising from neighbouring abdominal segments send tracheoles to the segment lacking its own branch, compensating for the lack of oxygen in the affected area. (B) A metabolically active ectopic organ (in red) was transplanted into the abdominal segment deprived from its own tracheal branch. The ectopic organ was immediately invaded by numerous tracheal projections, providing oxygen for metabolism of the organ. (C) *Rhodnius* larvae were placed in hypoxia and tracheal sprouting increased dramatically. (D) Local hypoxia was chemically created at a particular location of the *Rhodnius* abdomen (area in blue), and, as a consequence, many tracheal projections from nearby tracheal branches invade the hypoxic area. (E) In a *Drosophila* 3rd instar larva, the FGF homologue Branchless (green) was ectopically expressed in a single cell on the ectoderm. Tracheal terminal extensions projected by tracheal branches in the proximity are attracted towards the Branchless-expressing cell. (For interpretation of the references to color in this figure legend, the reader is referred to the web version of the article.)

Pioneering experiments carried out by Sir Vincent Wigglesworth in the 1950s revealed the hypoxia-mediated plasticity of insect tracheal system (Wigglesworth, 1954, 1983). By using the abdomen of *Rhodnius prolixus* as a model, Wigglesworth took advantage of the fact that individual tracheal branches provide oxygen to a particular abdominal hemisegment, and never cross the boundary to the neighboring or contralateral segment, which are supplied with oxygen by other tracheal branches. In a very simple yet elegant series of experiments, he managed to surgically severe the tracheal branch providing oxygen to half of the 4th tergite of the *Rhodnius* abdomen. He observed that tracheae coming from anterior and posterior segments, or from the contralateral side of the 4th segment, migrated into the oxygen-deprived hemisegment, (Fig. 1A) (Wigglesworth, 1954). Tracheoles were evidently endowed with the capacity to invade hypoxic areas of the body and, thereby, to compensate for the lack of oxygen.

To further challenge the system, Wigglesworth transplanted organs with a high rate of oxygen consumption, typically a *corpora allata* or a *corpora cardiaca*, into the oxygen-deprived hemisegment. In line with the above findings, nearby tracheae invaded the ectopic organ and supplied the extra-oxygen required for its high metabolic rate (Fig. 1B) (Wigglesworth, 1954). These experiments clearly showed the capacity of the tracheae to execute a compensatory response to oxygen deprivation.

In another series of experiments, entire insects were subjected to reduced oxygen tensions (typically between 5 and 10% O_2_). Animals challenged that way exhibited a more complex pattern of tracheal ramification (Fig. 1C), both in the thoracic and abdominal ganglia, as well as in the wings of adult *Rhodnius* (Wigglesworth, 1954). Finally, Wigglesworth developed a method to chemically induce strong local hypoxia in a small region of the larval abdomen. A few days afterwards, tracheal density in the hypoxic area was much higher than in the rest of the abdomen, which remained exposed to normal oxygen levels (Fig. 1D) (Wigglesworth, 1954). His discoveries on the adaptable nature of the tracheal system can be summarized best by his own words “*The tracheoles are by no means inert structures. They are capable of active migration into regions of deficient oxygenation*”. Among tracheal cells, he emphasized the role of the tracheal terminal cell: “*The reactive structure is presumably the tip of the tracheole* …”. Tracheal branches, therefore, possess the ability to *read* the oxygen requirements of the surrounding tissues, and to respond appropriately for an ensuing oxygen starvation in the tissue to be compensated (Wigglesworth, 1983).

Shortly after Wigglesworth's first description of the plasticity of the tracheal network in *Rhodnius* in response to diminished oxygen availability in 1954, Locke reported an even larger response of tracheal growth to varied oxygen tension in larvae of the hesperid butterfly *Calpodes* and the mealworm *Tenebrio* (Locke, 1958). To detect microscopical changes in tracheal growth, Wigglesworth and Locke both utilized cobalt naphthenate and the subsequent development of a red color compound upon reaction with 3,4-dinitroso-resorcinol within permeabilized tracheae (Wigglesworth, 1954; Locke, 1958). Small *Tenebrio* larvae were reared in hypoxia for up to 40 days. Even after one molt extra tracheation could be seen in some larvae, and marked changes occurred after three molts. Conversely, when *Tenebrio* larvae were raised in 50% oxygen, existing tracheae had grown in length only after three molts, while there was little, if any, growth of *de novo* formed tracheae and tracheoles (Locke, 1958).

Another leap forward came through the quantification of the tracheal hypertrophy response in hypoxic *Tenebrio* larvae by Loudon (1989). Similar to the observations of Locke, Loudon's work confirmed that some (dorsal, ventral, visceral), but not all (lateral longitudinal), tracheae show a significant hypertrophy at reduced oxygen level. This implied that tracheal plasticity is confined within the main branches that actually supply oxygen to tissues, whereas tracheae that primarily interconnect adjacent spiracles are less affected by changing levels of ambient oxygen. More recently, Henry and Harrison (2004) documented that, in addition to the developmentally plastic adaptations, there are also heritable changes in the dimensions of the dorsal tracheae (DT) of *Drosophila melanogaster* in response to multigenerational exposure to hypoxia and hyperoxia (Henry and Harrison, 2004). Rearing fruit flies for 5–6 generations in atmospheres of 10, 21 or 40% oxygen produced heritable effects in Dorsal Trunk diameters that were still observable after two generations in 21% O_2_ (Henry and Harrison, 2004). While these findings emphasize the tight regulation between tracheal morphology and physiology to ensure proper tissue *p*O_2_, both on acute and long-term time scales, the heritable components demand a more thorough analysis of the mechanistic underpinnings leading to tracheal plasticity in response to falling oxygen levels.

The availability of mutants and genetic tools that allow for gene manipulation in *Drosophila melanogaster* turned the fruit fly into an ideal model to take the early findings onto the genetic level. In fact, almost 50 years after Wigglesworth's hallmark discoveries in this field, Mark Krasnow and co-workers carried out a series of elegant experiments to investigate the genetic basis by which tracheae are attracted towards hypoxic areas (Jarecki et al., 1999). They showed that the expression of Branchless, the same FGF molecule that patterns *Drosophila* early tracheal development during embryogenesis (Sutherland et al., 1996), is regulated by oxygen levels during larval stages. Bnl protein levels rise when the larvae are exposed to 5% O_2_ (Jarecki et al., 1999), and accumulate in hypoxic tissues. These authors also managed to create a series of *Drosophila* strains with different levels of Bnl expression, and found a tight correlation between Bnl levels and the extent of tracheal ramification. Strikingly, they demonstrated that Bnl over-expression in an ectopic location of the larva is necessary and sufficient to direct the outgrowth of tracheal terminal branches towards that particular location (Fig. 1E) (Jarecki et al., 1999). In salivary glands, normally devoid of tracheae, miss-expression of Bnl led to the colonization of the target tissue by tracheal branches. In another series of experiments, they showed the striking capacity of Bnl to act as a tracheal chemo-attractant by expressing Bnl in individual cells; this was enough to attract tracheal extensions from terminal cells located at a distance of several cell diameters (Fig. 1E) (Jarecki et al., 1999).

From these studies, the immanent question was, *where* and *how* the sensing of dropping *p*O_2_ takes place to allow for changes in gene expression that result in tracheole extension in insects. Elucidation of this cellular oxygen sensing pathway required several more years. It was first unraveled in nematodes and mammals, and soon afterwards, also in *Drosophila*.

## 5 The oxygen sensing pathway

During the early 1970s, historical experiments by Judah Folkman and colleagues led to the conclusion that growing tumours are capable of inducing an angiogenic response from pre-existing blood vessels (Folkman, 1974). Subsequently, an abundance of efforts was dedicated to understand the nature of this induction. Progress of this work culminated in the identification of the Vascular Endothelial Growth Factor (VEGF) as a potent secreted angiogenic signal *in vivo* (Leung et al., 1989). Soon afterwards, it was revealed that VEGF was strongly induced by oxygen deprivation, a typical feature of many growing tumors (Shweiki et al., 1992). The anticipated master regulator of the transcriptional response to hypoxia (Maxwell et al., 1993) was eventually isolated as the so-called Hypoxia Inducible Factor 1 (HIF-1) by Wang and Semenza in 1995 (Wang et al., 1995; Wang and Semenza, 1995). The discovery of HIF-1, and the identification of VEGF as one of its transcriptional targets (Maxwell et al., 1997), ultimately allowed building the current model for tumor angiogenesis: (i) A massive cell growth in a tumor rapidly provokes hypoxia, (ii) HIF induces the expression of VEGF by tumor cells, and (iii) VEGF is the angiogenic signal that allows the recruitment of new blood vessels by the tumor.

**Fig. 2:**
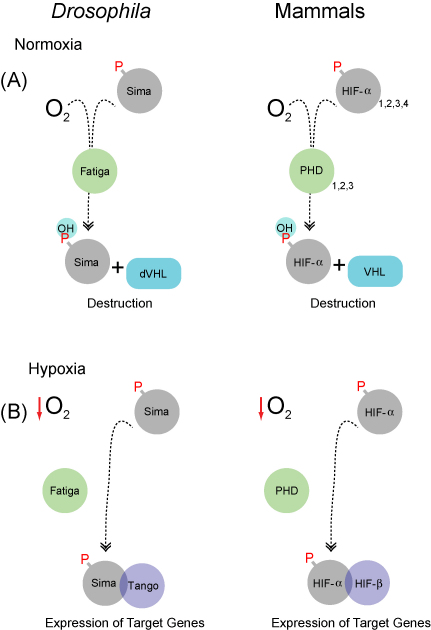
Oxygen-dependent regulation of the stability of HIF-α proteins. Sima (in *Drosophila*) or HIF-α (in mammals) are hydroxylated at specific prolyl residues in an oxygen-dependent manner, in a reaction catalyzed by specific prolyl hydroxylases (“Fatiga” in *Drosophila*; PHDs in mammals). (A) Hydroxylation of specific prolines of HIF-α proteins in normoxia enables interaction with the Von Hippel Lindau (VHL) tumor suppressor factor, which is the substrate recognition subunit of an E3 ubiquitin ligase enzyme. Interaction with VHL leads to HIF-α proteasomal degradation. (B) In hypoxia, VHL fails to interact with HIF-α due to the inhibited prolyl hydroxylation, resulting in stabilization of the protein, subsequent dimerization with the HIF-β subunit (“Tango” in *Drosophila*), and induction of HIF-dependent gene expression.

HIF proteins are bHLH-PAS α/β heterodimers (Wang et al., 1995), thus belonging to the same protein family (bHLH-PAS transcription factors) that also includes Trachealess in *Drosophila* (Isaac and Andrew, 1996; Wilk et al., 1996). While the β-subunit of HIF-1 is constitutively present, the stability and transcriptional activity of the α-subunit is regulated by oxygen levels. HIF-1α is rapidly degraded when oxygen is available (“normoxia”) (Fig. 2A) (Pugh et al., 1997; Huang et al., 1998). In contrast, minutes-to-hours of low oxygen stabilize the HIF-1α protein and render it transcriptionally competent. Now, the factor translocates into the nucleus where it heterodimerizes with HIF-1β. The α/β complex binds to specific DNA recognition motifs, so-called hypoxia response elements (HREs) (Maxwell et al., 1993; Firth et al., 1995), from where it induces the expression of target genes that mediate cellular and physiological adaptations to hypoxia (Fig. 2B).

The stability and transcriptional activity of HIF-α factors depends on the action of oxygen-dependent prolyl hydroxylases, known as PHDs (“Prolyl Hydroxylase Domain” containing polypetides) (Bruick and McKnight, 2001; Epstein et al., 2001), and an asparaginyl hydroxylase (FIH-1) (Lando et al., 2002, 2003), respectively. These enzymes (in mammals: PDH-1 to PDH-3 + FIH-1) utilize molecular oxygen as a co-substrate to catalyze either the hydroxylation of key prolyl residues, located in the central Oxygen Dependent Degradation (ODDD) domain of HIF-α proteins (i.e. PHDs) (Ivan et al., 2001; Jaakkola et al., 2001), or of a single asparaginyl residue housed within the C-terminal transactivation domain (C-TAD) of the factor (i.e. FIH-1) (Lando et al., 2002). Upon hydroxylation of the prolyl residues of the ODDD by PHD1-3, HIF-α subunits are efficiently recognized by the Von Hippel Lindau (VHL) tumor suppressor factor, a component of an E3 ubiquitin ligase complex that targets them to proteasomal destruction (Fig. 2) (Maxwell et al., 1999). Hydroxylation of the single C-TAD asparagine by FIH-1, on the other hand, inhibits the interaction of HIF-α subunits with co-activating factors (e.g. p300) (Lando et al., 2002). While this asparagine hydroxylation is still permissive for the factor's binding to DNA, it effectively shuts down any HIF-driven transactivation of HRE-flanked target genes, since the co-activator is crucial to link the HIF complex with the RNA polymerase II driven basal transcription machinery. Given that PHDs and FIH-1 have an absolute requirement of dioxygen for their catalytic activity, and, based on the fact that their Km for oxygen is around the physiological range of oxygen tension, these hydroxlyases are considered *bonafide* oxygen sensors of the cell (Kaelin and Ratcliffe, 2008).

In *Drosophila melanogaster*, the basic machinery for sensing and responding to hypoxia is very similar to the one of mammalian species, although with less redundancy (Gorr et al., 2006; Romero et al., 2007). Whereas three HIF-α subunits occur in mammals, there is just one HIF-α in the fruit fly, which is encoded by the gene *similar* (*sima*) (Nambu et al., 1996; Bacon et al., 1998). Likewise, there is just one PDH oxygen sensor in the fly, encoded by the gene *fatiga* (*fga*), that controls Sima half-life as a function of *p*O_2_ (Fig. 2) (Lavista-Llanos et al., 2002; Centanin et al., 2005; Dekanty et al., 2005). Interestingly, the fruit fly genome does neither seem to contain a structural (functional?) FIH-1 homologue, nor are there conserved asparagine residues present near the Sima C-terminus in the fly. The branchiopod crustacean *Daphnia magna* or the nematode *C. elegans* also seem to lack the asparagine hydroxylation control feature, it therefore appears that prolyl-hydroxylation and α-subunit degradation phylogenetically preceded the transcriptional inactivation of HIF under high *p*O_2_ via FIH-homologous catalysis (our unpublished observations).

*Drosophila* embryos homozygous for any of the *fga* mutant alleles exhibit constitutive accumulation of Sima protein, even in normoxia (Centanin et al., 2005). Sima levels in embryos exposed to hypoxia are actually much lower than those of *fga* mutants. Interestingly, all *fga* mutant alleles are lethal at different developmental stages. The availability of a series of mutants for both *fatiga* and *sima,* together with the lack of genetic redundancy of the hypoxia-responsive machinery in the fruit fly, provided a good opportunity to address whether Sima/HIFα represents the only hydroxylation target of Fatiga/PHD. By generating *fatiga* *sima* double mutant flies, the two phenotypes – constitutive response to hypoxia and lethality – were rescued, and individuals were now able to reach adult stages (Centanin et al., 2005). The fact that *fga sima* double mutants are able to develop to adulthood suggests that the oxygen sensing machinery is a dispensable function under favorable environmental conditions.

However, *Drosophila* *fatiga* mutants exhibit a striking extra-sprouting phenotype of the tracheal system, which was even more pronounced than that of larvae grown in hypoxia (Centanin et al., 2008; Moberg and Mortimer, 2009). When *fatiga* *sima* double mutants were analyzed, tracheal ramification remained at levels of control larvae, suggesting that tracheal extra-sprouting in *fga* mutants results from the over-accumulation of Sima (Centanin et al., 2008).

## 6 An autonomous role of the tracheal system in the branching response to low oxygen levels

**Fig. 3:**
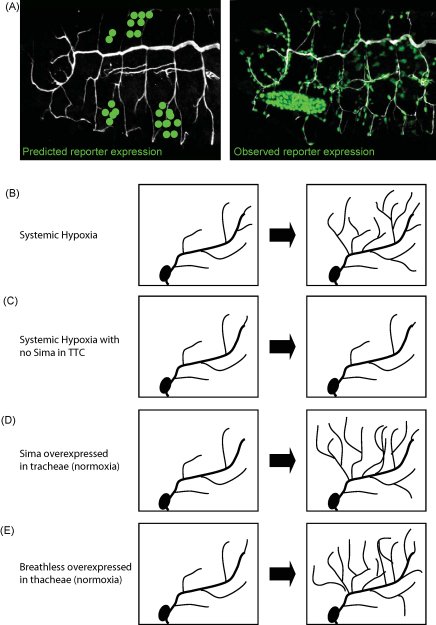
Transcriptional cell-autonomous response to hypoxia of *Drosophila* tracheal cells. (A) *Left panel*: Photograph of a *Drosophila* normoxic embryo transgenic for a GFP hypoxia-inducible transcriptional reporter. The GFP reporter is not expressed in conditions of oxygen availability; the green dots indicate the position in which the reporter is expected to be expressed in hypoxia, according to the models that account for mammalian angiogenesis (i.e. in target tissues). *Right panel*: In embryos exposed to hypoxia, the HIF-dependent reporter is induced in tracheal cells, and not in target tissues, as would predict by the angiogenesis-born model. (B) In *Drosophila* larvae exposed to hypoxia, tracheal terminal cells (TTCs) generate extra-ramifications. (C) In larvae exposed to hypoxia, the sprouting response schematized in (A) does not occur in TTCs that are mutant for Sima. (D) Over-expression of Sima, specifically in tracheal cells, is sufficient to induce tracheal terminal cell (TTC) sprouting in a normoxic larva. (E) Tracheal over-expression of the FGF receptor Breathless is also sufficient to promote tracheal extra-sprouting in normoxia.

Since the above experiments clearly implicated the prolyl-hydroxylase Fatiga and the HIF-α protein Sima in controlling tracheal extra-sprouting during hypoxia, we generated a series of hypoxia-responsive transgenic reporters to further analyse the tracheal response to low oxygen levels in the fruit fly (Lavista-Llanos et al., 2002). Typically, the HRE-studded promoter of the murine *lactate dehydrogenase* (*ldh*) gene, a gene known to be induced by HIF at low oxygen levels, was used to drive the expression of GFP or other reporter proteins, such as LacZ. This useful tool contributed to analyzing the dynamics of the hypoxic response in vivo (Lavista-Llanos et al., 2002). Based on the expression of the transgenic reporter in graded hypoxia, assessment of organs or cell types that induce hypoxia-dependent gene expression with high sensitivity was made possible. The expected scenario, according to the models that account for angiogenesis, predicted that hypoxia is first sensed in target tissues (Fig. 3A), which would then turn-on Sima-dependent induction of *branchless*, leading to tracheal extra-sprouting. Surprisingly, this was not the case (Lavista-Llanos et al., 2002; Centanin et al., 2008). Tracheal cells actually express the hypoxia-responsive reporter with higher sensitivity than any other cell type in the body (Fig. 3A). More precisely, the tracheal terminal cells (TTCs) are, within the tracheal system, the ones most sensitive to hypoxia (Centanin et al., 2008). Therefore, TTCs (those “*cells at the tip of each tracheal branch*” described by Wigglesworth) (Wigglesworth, 1954) possess three main and inter-related properties which enable them to function as cellular O_2_ sensors: (a) They are the only cells of the tracheal system with a capacity to deliver oxygen to target tissues. (b) They are the only ones with a capacity to sprout-out cytoplasmic projections under conditions of low oxygen availability, and (c) They induce hypoxia-dependent gene expression with higher sensitivity than any other cell in the organism.

Several questions are immediately inspired by the above findings: How do oxygen-sensing genes mediate TTC sprouting? Is TTC sprouting a cell autonomous response or does it rather reflect a response to stimuli generated by surrounding tissues?

Since the hypoxic reporter was first induced in tracheal cells rather than in target tissues (Fig. 3A) (Centanin et al., 2008), induction of tracheal specific genes in hypoxia likely reflects a primary transcriptional response of the organism. We determined that the FGFR homologue Btl is a key target of Sima in response to hypoxia. Moreover, forced over-expression of either Sima or Breathless in TTCs provoked a tracheal sprouting phenotype highly reminiscent to that observed in hypoxia, (Fig. 3D and E) (Centanin et al., 2008). Thus, up-regulation of Sima or Btl in TTCs is sufficient to induce tracheal sprouting, whereas, suppression of the expression of Sima specifically in TTCs totally prevented tracheal sprouting (Fig. 3B and C). Thus, up-regulation of Sima in TTCs is necessary and sufficient for promoting tracheal sprouting in response to hypoxia (Fig. 3C and D) (Centanin et al., 2008).

**Fig. 4:**
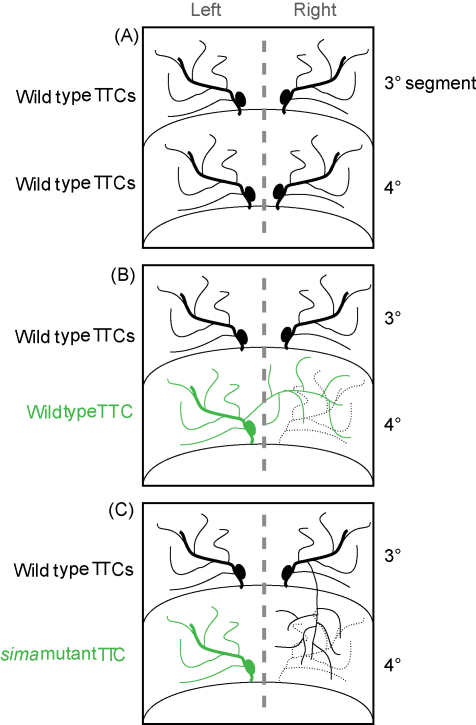
Compensatory response of the *Drosophila* tracheal system in response to a missing tracheal terminal cell (TTC). (A) One tracheal terminal cell (TTC) occurs in each dorsal branch. There are two dorsal branches per segment (one on the left; the other one on the right). (B) TTCs are stochastically lost in *Drosophila* larvae; in the example depicted in the scheme, the TTC corresponding to the dorsal branch of the 4th segment is missing. The TTC from the contralateral hemisegment extends cytoplasmic projections that cross the dorsal midline, invading the region lacking its own dorsal branch. (C) If the TTC contralateral to the missing branch is mutant for Sima, it fails to execute the compensatory response (i.e. it does not invade the hypoxic contralateral hemisegment). Instead, a TTC from the adjacent segment (the 3rd segment in the example) extends projections that provide oxygen to the region lacking its own tracheal branch.

Each *Drosophila* tracheal dorsal branch has only one TTC (there is one dorsal tracheal branch per hemisegment), and ramifications coming from TTCs neither cross the dorsal midline nor invade neighbouring segments (Fig. 4A). Sporadically, as a random developmental event, one or more TTCs fail to differentiate in *Drosophila* larvae. In response to the lack of a given dorsal branch TTC, the TTC corresponding to the contralateral dorsal branch of the same segment sprouts-out ramifications that invade the oxygen-starved area, thereby compensating for the lack of oxygen at the hemisegment lacking its own TTC (Fig. 4B) (Jarecki et al., 1999; Centanin et al., 2008). This developmental response to hypoxia of *Drosophila* 3rd instar larval tracheae is equivalent to the compensatory response observed by Wigglesworth in the tracheal system of *Rhodnius* abdomens, after surgical elimination of the tracheal branch of the 4th tergite (see Fig. 1A) (Wigglesworth, 1954). Interestingly enough, this compensatory response to a missing TTC in the *Drosophila* larva fails to occur when the remaining (contralateral) TTC is mutant for Sima. In this case, wild type TTCs coming from neighbouring segments project cytoplasmic extensions to alleviate the oxygen starvation in the affected hemisegment (Fig. 4C) (Centanin et al., 2008). Altogether, these experiments reveal that TTCs respond to hypoxia in an autonomous manner by sending cytoplasmic projections to tissues with poor oxygen supply. This autonomous response depends on the accumulation of Sima protein in the TTC, which in turn induces transcriptional up-regulation of the FGFR Btl in this cell. Up-regulation of the Breathless receptor probably increases sensitivity of TTCs to available levels of the FGF homologue Bnl, which is transcriptionally induced locally in target tissues with poor oxygenation (Centanin et al., 2008). Accumulation of the Bnl cue in the target tissues provides the necessary directionality to the outgrowth of tracheal branches (Fig. 1E) (Jarecki et al., 1999).

Regarding these characteristics, insect TTCs are analogous to the “Tip cells” of the mammalian vascular system. These cells too are located *at the tip* of a growing blood capillary and can sense gradients of different guidance molecules. Through the expression of VEGF receptors (among others), they are responsible for guiding new blood vessels towards a source of VEGF. Therefore, tip cells play a crucial role during angiogenesis, leading the sprouting vessels directly towards the hypoxic interior of the growing tumor (Holderfield and Hughes, 2008; Sainson et al., 2008).

Do mammalian endothelial cells undergo a cell-autonomous response during angiogenesis, analogous to the one of TTCs during tracheal sprouting? Recent studies in mice suggest that this may well be the case. Randall Johnson and colleagues reported in a mouse model that the endothelial cell-selective knockdown of HIF-1α markedly impairs tumor angiogenesis (Tang et al., 2004). This clearly demonstrates a requirement for oxygen responsive genes within the vascular system and not just in the target tissues. Analogies between TTCs and endothelial tip cells seem to extend even further: Differentiation of TTCs during *Drosophila* embryonic development is induced by the localized expression of the *Drosophila* Serum Response Factor (dSRF) (Affolter et al., 1994; Guillemin et al., 1996). Likewise, we now know that the differentiation of mammalian tip cells during angiogenesis depends on mammalian SRF (Franco et al., 2008). Further detailed research of the physiology of mammalian tip cells will reveal to what extent the cell autonomous behaviour of these cells is relevant to angiogenesis, and to what extent these cells are mere responders to guidance cues emanating from surrounding tissues.
